# MicroRNA-223 negatively regulates the osteogenic differentiation of periodontal ligament derived cells by directly targeting growth factor receptors

**DOI:** 10.1186/s12967-022-03676-1

**Published:** 2022-10-11

**Authors:** Zheng Zhang, Minghui Wang, Youli Zheng, Yanmei Dai, Jiashu Chou, Xiaowei Bian, Pengcheng Wang, Changyi Li, Jing Shen

**Affiliations:** 1 Tianjin Stomatological Hospital, School of Medicine, Nankai University, Tianjin, 300041 China; 2 State Key Laboratory of Natural and Biomimetic Drugs, Peking University, Beijing, 100191 China; 3 Tianjin Key Laboratory of Oral and Maxillofacial Function Reconstruction, Tianjin, 300041 China; 4 The School and Hospital of Stomatology, Tianjin Medical University, Tianjin, 300070 China; 5 Department of Stomatology, Beijing Shijitan Hospital, Capital Medical University, Beijing, 100038 China

**Keywords:** Periodontitis, MicroRNA-223, Periodontal ligament stem cells, Osteogenesis

## Abstract

**Background:**

MicroRNA (miRNA) is accepted as a critical regulator of cell differentiation. However, whether microRNA-223 (miR-223) could affect the osteogenic differentiation of periodontal ligament (PDL)-derived cells is still unknown. The aim of this study was to explore the mechanisms underlying the roles of miR-223 in the osteogenesis of PDL-derived cells in periodontitis.

**Methods:**

Microarray analysis and real-time polymerase chain reaction (RT-PCR) were used to identify difference in miR-223 expression pattern between healthy and inflamed gingival tissue. The target genes of miR-223 were predicted based on Targetscan and selected for enrichment analyses based on Metascape database. The gain-and loss-of-function experiments were performed to discuss roles of miR-223 and growth factor receptor genes in osteogenic differentiation of PDL-derived cells. The target relationship between miR-223 and growth factor receptor genes was confirmed by a dual luciferase assay. Osteogenic differentiation of PDL-derived cells was assessed by Alizarin red staining, RT-PCR and western blot detection of osteogenic markers, including osteocalcin (OCN), osteopontin (OPN) and runt-related transcription factor 2 (Runx2).

**Results:**

MiR-223 was significantly increased in inflamed gingival tissues and down-regulated in PDL-derived cells during osteogenesis. The expression of miR-223 in gingival tissues was positively correlated with the clinical parameters in periodontitis patients. Overexpression of miR-223 markedly inhibited PDL-derived cells osteogenesis, which was evidenced by reduced Alizarin red staining and osteogenic markers expressions. Furthermore, two growth factor receptor genes, including fibroblast growth factor receptor 2 (FGFR2) and transforming growth factor beta receptor 2 (TGFβR2), were revealed to be direct targets of miR-223 and shown to undergo up-regulation in PDL-derived cells during osteogenesis. Moreover, suppression of FGFR2 or TGFβR2 dramatically blocked PDL-derived cells osteogenic differentiation.

**Conclusions:**

Our study provides novel evidence that miR-223 can be induced by periodontitis and acts as a negative regulator of PDL-derived cells osteogenesis by targeting two growth factor receptors (TGFβR2 and FGFR2).

**Supplementary Information:**

The online version contains supplementary material available at 10.1186/s12967-022-03676-1.

## Background

Periodontitis is a highly prevalent infectious disease, characterized by gingival inflammation, resorption of alveolar bone and tooth loosening or even loss [1]. It is one of the top ten highly prevalent diseases worldwide and the main cause of adult tooth loss [2]. Periodontitis is thought to be caused by periodontal microbiota and related host immune response, which result in the damage of periodontal tissue [3]. Periodontal ligament (PDL)-derived cells possess superior abilities for differentiating into bone, cementum and periodontal ligament, and play a vital role in the restoration of periodontal tissues [4]. However, inflammatory microenvironment leads to the dysfunction of PDL-derived cells, which greatly inhibits their capacity for periodontal tissue regeneration [5]. Thus, modulating the biological functions of PDL-derived cells during an inflammatory challenge is one of the goals of treating periodontitis.

As small noncoding RNA molecules, microRNAs (miRNAs) participate in the progress of periodontitis through the negative regulation of target genes [6]. In the field of cell differentiation, several miRNAs have been proven to regulate the osteogenesis of PDL-derived cells and become key regulators of bone remodeling in periodontitis [4]. Previous miRNA microarray analyses, conducted by Stoecklin-Wasmer et al. [7] and Yorimasa et al. [8], have suggested that microRNA-223 (miR-223) is one of the most highly expressed miRNAs in periodontitis tissues. Periodontitis is correlated with increased levels of some inflammatory mediators in serum and gingival crevicular fluid (GCF) [9]. A recent research has reported that miR-223 is significantly overexpressed in both serum and GCF of periodontitis patients [2]. In previous study, miR-223 was shown to regulate osteogenesis of bone marrow-derived mesenchymal stem cells (BMSCs) of mouse [10] and human [11]. However, it remains unclear the expression of miR-223 changes after osteogenic induction in PDL-derived cells and, if so, what its regulation function may be.

MiRNAs primarily function as gene regulators and can act directly on their target gene, which in turn regulates gene expression [2]. Our bioinformatic analysis prior to our investigation identified two important growth factor receptor genes to be potentially targeted by miR-223, including fibroblast growth factor receptor 2 (FGFR2) and transforming growth factor beta receptor 2 (TGFβR2). FGFR2 functions as a fibroblast growth factor (FGF) receptor, transducing FGF signals to other signaling cascades [12]. It has been demonstrated to play pivotal roles in osteoblast differentiation [13]. TGFβR2, a receptor of the transforming growth factor beta (TGF-β), is also a major regulator of osteogenesis and bone repair [14]. Additionally, members of the FGFs and TGFβ families are known to be essential for osteoblast differentiation [15]. Therefore, we speculated that FGFR2 and TGFβR2 may participate in the action of miR-223 in PDL-derived cells osteogenesis. Since miR-223 plays a significant role in the osteogenic differentiation of stem cells, and growth factor receptors (TGFβR2 and FGFR2) are forecasted to be the target genes of miR-223 by bioinformatics analysis, we hypothesized that miR-223 may participate in the osteogenic differentiation of PDL-derived stem cells by regulating growth factor receptors. The aim of this study was to examine whether and how miR-223 affects the osteogenic differentiation of PDL-derived cells.

## Material and methods

### Expression profiles analysis of miRNA

The miRNA expression profiles (GSE54710) of periodontitis were downloaded from the Gene Expression Omnibus (GEO) database (http://www.ncbi.nlm.nih.gov/geo/). The expression profiles comprised 200 gingival tissue samples, including 98 chronic periodontitis samples, 30 chronic periodontitis control samples, 61 aggressive periodontitis samples and 11 aggressive periodontitis control samples. Differentially expressed miRNAs (DE-miRNAs) between periodontitis and control were identified using GEO2R online tool (https://www.ncbi.nlm.nih.gov/geo/geo2r/). A threshold was set at |log_2_ fold change|> log_2_ 1.5 and *P*-value < 0.05, from which the periodontitis-associated DE-miRNAs were selected.

### Prediction of miR-223 targets and protein–protein interactions (PPI) network construction

The target genes of miR-223 were predicted using Targetscan (http://www.targetscan.org). These genes were imported into the STRING database (http://www.string-db.org/) to obtain the PPI network and then visualized with Cytoscape. Subsequently, Community Clustering (Glay) was applied to cluster the PPI network and obtain the key clusters that influence periodontitis. The clusters in which the target genes of miR-223 were mainly concentrated were defined as key clusters.

### Functional and pathway enrichment analysis of miR-223 target genes

To determine the biological processes and pathways of the target genes of miR-223, these genes were imported into the Metascape database (https://metascape.org/gp/index.html#/main/step1) for Gene Ontology (GO) biological processes and Kyoto Encyclopedia of Genes and Genomes (KEGG) pathways enrichment analysis. *P*-value < 0.05 was considered as statistically significant.

### Patient and samples collection

A total of 40 subjects, including 20 healthy controls and 20 periodontitis patients, were enrolled in our study. The study was approved by the ethics committee of Tianjin Stomatological Hospital. Periodontitis was diagnosed as probing depth (PD) > 3 mm, attachment level (AL) > 2 mm, and bleeding on probing. The control group included patients with no signs of gingival inflammation, with PD ≤ 3 mm, AL ≤ 2 mm, and no bleeding on probing. Patients with disease known to affect periodontitis were excluded. Patients had medications that affect periodontitis within half a year were also excluded. All subjects underwent the clinical examination, including plaque index (PI), gingival index (GI), bleeding index (BI), PD and AL during their surgical procedure. Gingival tissues were obtained, frozen by liquid nitrogen and stored at -80 °C for further analysis.

### Isolation and cell cultures of PDL-derived cells

The periodontal ligament tissues were collected from 30 teeth, which were premolars or third molars extracted from 20 healthy donors aged 14–23 years. The tissues were cut into small pieces, and digested in 3 mg/ mL collagenase type I (Sigma-Aldrich Corp., USA) and 4 mg/ mL dispase II (Sigma-Aldrich Corp., USA) for 30 min. Cell suspensions of periodontal ligament tissues were suspended in complete medium containing α-MEM, 10% fetal bovine serum (FBS, Gibco, USA), 100 ug/mL of streptomycin, and 100 units/ mL of penicillin (Gibco, USA). Cells were cultured at 37 °C with 5% CO_2_, and the medium was refreshed every other day. PDL-derived cells used in our study were at passage 3–5.

### Flow cytometry

It has been confirmed that PDL-derived stem cells showed mesenchymal stem cell (MSC)-like characteristics such as cell surface marker expression (CD90 + , CD73 + , CD44 + , CD45 − , and CD34 −) [4, 16, 17]. To confirm stem cell characteristics of PDL-derived cells isolated with this method, flow cytometry was used to evaluate cell surface markers. Briefly, PDL-derived cells (1 × 10^6^ cells) were digested by 0.25% trypsin and resuspended in PBS. Anti-human stem cell surface-labeled antibodies including CD34, CD44, CD45, CD73 and CD90 (all from BD Pharmingen, USA) was added to cell suspension. The samples were incubated at 4 °C in the dark for 20 min. Then the cells were washed with PBS twice and resuspended in 100 μL of PBS for flow cytometry analysis.

### Osteogenic differentiation of PDL-derived cells

PDL-derived cells were cultured in 6-well plates at 1 × 10^5^ cells/well, and induced by osteogenic differentiation medium, which was composed of a culture medium supplemented with 50 mg/L ascorbic acid, 10 mM/L dexamethasone, and 10 mM /L β-glycerophosphate (Sigma, USA). Cells were treated for 7 and 14 days, and the medium was refreshed every 3 days.

### Cell transfection

PDL-derived cells were seeded into 6-well plates at 1 × 10^5^ cells/well, and transfected at 40–50% confluence. MiR-223 mimic and mimic control (miR-223 NC) were synthesized by Sangon (Shanghai, China), and transfected into PDL-derived cells using Lipofectamine 2000 (Invitrogen, USA). The control siRNA (Si-NC) and siRNA duplexes specific for TGFβR2 and FGFR2 were purchased from RiboBio (Guangzhou, China). SiRNAs were transfected into PDL-derived cells using riboFECT™ CP (RiboBio, China)) according to the manufacturer’s instructions.

### Real-time polymerase chain reaction (RT-PCR)

This research isolated total RNA using MiniBEST Universal RNA Extraction Kit (TaKaRa, Japan) based on the manufacturer’s protocols. The cDNA of miRNA was synthesized by All-in-One™ miRNA First-Strand cDNA Synthesis Kit (GeneCopoeia, USA). All-in-One™ miRNA qPCR Kit (GeneCopoeia, USA) was used for quantitative PCR. U6 was used for normalization. The cDNA of mRNA was synthesized by PrimeScriptTM RT reagent kit (TaKaRa, Japan). TB GreenPremix Ex Taq™II (TaKaRa, Japan) was then used for quantitative PCR. GAPDH was used for normalization. The primer sequences used in the experiment were shown in Additional file 1: Table S1.

### Western blotting

Total protein was obtained from PDL-derived cells by the protein lysate (Beyotime, China), and centrifuged with 12000 rpm for 10 min. Protein samples were then electrophoresed on 10% SDS-PAGE gel, transferred onto PVDF membranes (Millipore, USA), which was blocked with 5% non-fat milk. The primary antibody was incubated overnight at 4 °C and the secondary antibody was incubated for 1 h at room temperature. ECL solution was then prepared, and the bands on the membranes were scanned. The following primary antibodies were TGFβR2 (Abcam, UK), FGFR2 (Beyotime, China), osteopontin (OPN; Abcam, UK), osteocalcin (OCN; Abcam, UK), Runt-related transcription factor-2 (Runx2; Abcam, UK) and GAPDH (proteintech, USA).

### Alizarin red staining

The PDL-derived cells were cultured in an osteogenic medium for 7 or 14 days. We then fixed the cells in 4% paraformaldehyde for 15 min at room temperature. The cells were washed 3 times with PBS, and stained with 1% Alizarin Red S (Solarbio, China) according to the manufacturer's instructions. Finally, the cells were washed and photographed.

### Dual luciferase reporter gene assay

The 3’-untranslated region (3′UTR) of TGFβR2 and FGFR2 sequences containing the binding sites of miR-223 was synthesized. Wild type (WT) 3′UTR plasmids and mutant type (MUT) 3’UTR plasmids were constructed, and then transfected into PDL-derived cells with miR-223 and control plasmids respectively. The luciferase activity was assayed using a Dual-Luciferase Reporter Assay Kit (Promega, USA) 48 h post-transfection according to the manufacturer’s instructions.

### Statistical analysis

All data above are expressed as the means ± standard deviation. The t test was applied for comparisons between two groups, while one-way ANOVA with Bonferroni correction for multi-groups. Pearson’s correlation was used to study the relation between miR-223 level and clinical periodontal indexes. Receiver operating characteristic (ROC) analysis was used to study the effect of miR-223 on the disease. All statistical analyses were performed using SPSS13.0 (SPSS Inc., Chicago, IL, USA). *P*-value < 0.05 was considered statistically significant. All experiments were repeated at least three times, and representative experiments are shown.

## Results

### Identification of DE-miRNAs

**Fig. 1:**
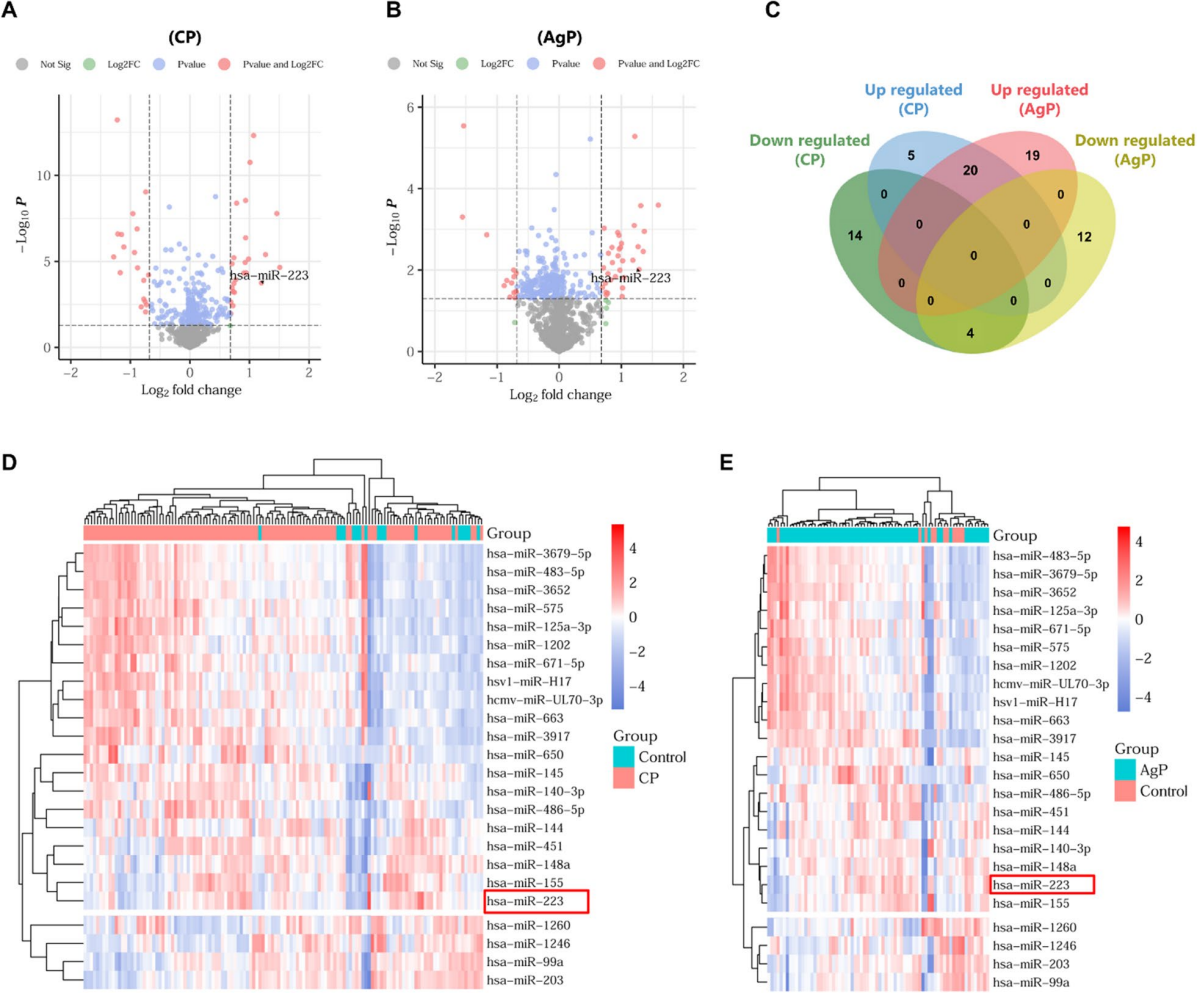
Identification of differentially expressed miRNAs (DE-miRNAs) in periodontitis. **A** Volcano plot of DE-miRNAs in chronic periodontitis (CP) and normal gingival tissue samples. **B** Volcano plot of DE-miRNAs in aggressive periodontitis (AgP) and normal gingival tissue samples. **C** Venn diagrams of overlapping DE-miRNAs between CP and AgP data. **D** Heatmap of DE-miRNAs in CP and normal gingival tissue samples. **E** Heatmap of DE-miRNAs in AgP and normal gingival tissue samples

Using the GEO database, we obtained 43 DE-miRNAs (25 upregulated and 18 downregulated) between chronic periodontitis and control gingival samples (Fig. 1A), and 55 DE-miRNAs (39 upregulated and 16 downregulated) between aggressive periodontitis and control gingival samples (Fig. 1B). As shown in Fig. 1C, 20 upregulated and four downregulated DE-miRNAs were commonly appeared in two types of periodontitis. Among these 24 DE-miRNAs, miR-223 was significantly upregulated in periodontitis (Fig. 1D, E).

### MiR-223 expression in the inflamed gingival tissues

**Fig. 2:**
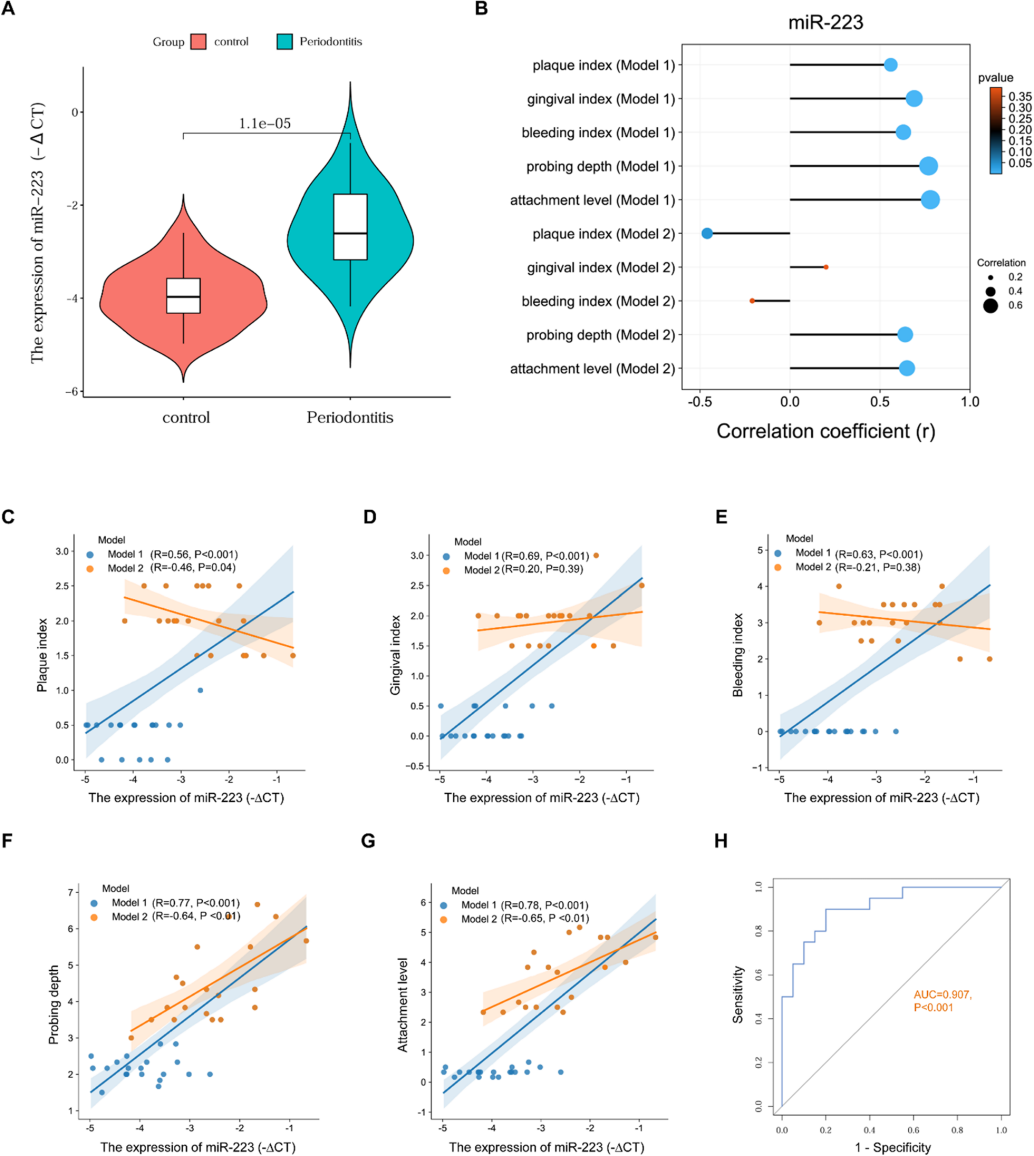
The expression of miR-223 in the inflamed gingival tissues. **A** RT-qPCR quantification of miR-223 expression in gingival tissue samples. **B** The correlations of miR-223 expression with clinical periodontal indexes. **C**–**G** Correlation scatter plots between miR-223 expression and plaque index (**C**), gingival index (**D**), bleeding index (**D**), probing depth (**F**) and attachment level (**G**). **H** The receiver operating characteristic (ROC) curve of miR-223 in periodontitis. Model 1, the correlation analysis based on all subjects, including periodontitis patients and control subjects. Model 2, the correlation analysis based on periodontitis patients

A significant increase of miR-223 expression in the inflamed gingival samples was observed when compared to control samples (Fig. 2A). The correlation analysis based on all subjects, including periodontitis patients and control subjects, demonstrated that miR-223 was positively correlated with PI, GI, BI, PD and AL. However, miR-223 only showed a positive correlation with PD and AL in periodontitis patients (Fig. 2B–G). ROC curve analysis revealed that miR-223 in gingival tissues had a significant area under the curve (AUC) value of 0.907 (P < 0.001) (Fig. 2F). The clinical characteristics of healthy controls and periodontitis patients are shown in Additional file 1: Table S2.

### MiR-223 expression in PDL-derived cells

**Fig. 3:**
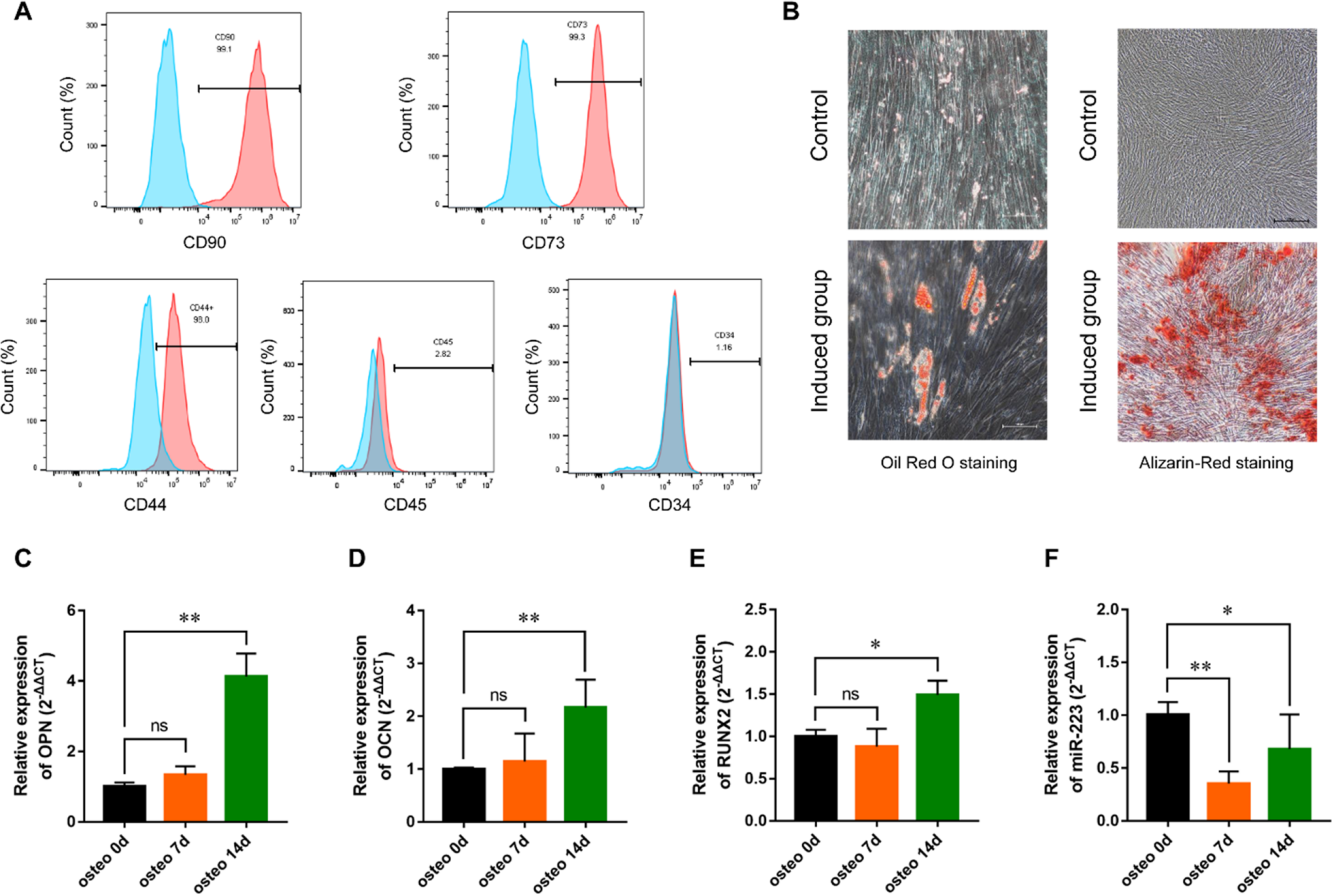
Characterization of periodontal ligament (PDL)-derived cells and miR-223 expression in PDL-derived cells. **A** Flow cytometric analysis of surface markers of PDL-derived cells. **B** Representative images of adipogenic differentiation and osteogenic differentiation. **C**–**E** RT-qPCR quantification of osteogenic-specific genes (OCN, OPN and Runx2) expressions after osteogenic induction. **F** RT-qPCR quantification of miR-223 expression in PDL-derived cells cultured in osteogenic induction medium. All experiments were performed in triplicate, and the bars represent the mean ± SD

Flow cytometry analyses showed that PDL-derived cells positively expressed CD90, CD73 and CD44, and were negative for CD34 and CD45 (Fig. 3A). After induction, PDL-derived cells demonstrated the characteristics of multipotent differentiation (Fig. 3B). Following osteogenic induction, the expressions of osteogenic marker genes (OCN, OPN and Runx2) in PDL-derived cells were found to be markedly increased (Fig. 3C). Additionally, miR-223 expression was significantly down-regulated in PDL-derived cells cultured in osteogenic induction medium (Fig. 3D).

### MiR-223 inhibits PDL-derived cells osteogenesis

**Fig. 4:**
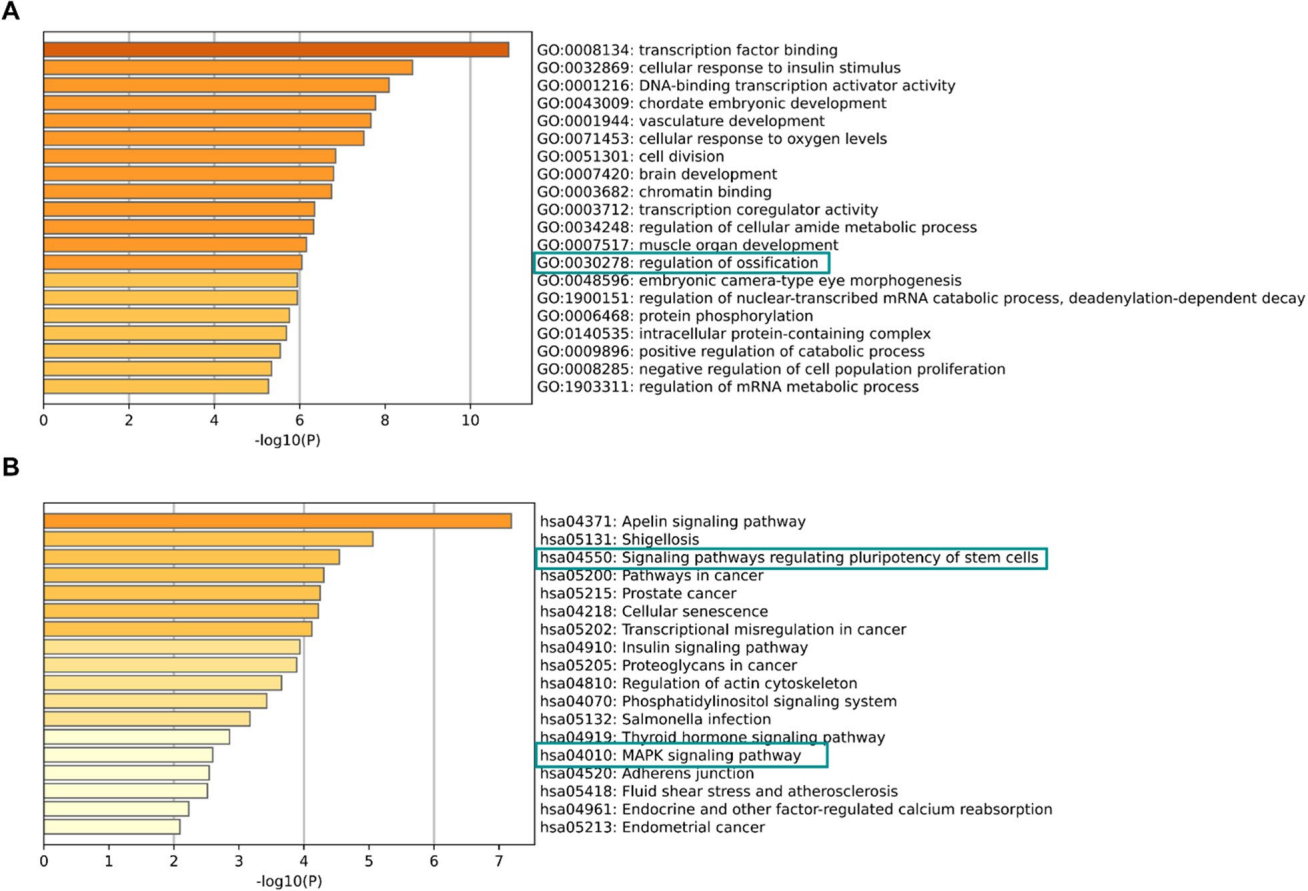
Functional and pathway enrichment analysis of miR-223 target genes. **A** TOP20 GO terms of miR-223 target genes. **B** TOP20 KEGG pathway of miR-223 target genes

GO enrichment analysis showed that miR-223 target genes were significantly enriched in regulation of ossification (Fig. 4A). KEGG pathway enrichment analysis revealed that target genes of miR-223 were significantly enriched in osteogenic differentiation related signaling pathways, such as signaling pathways regulating pluripotency of stem cells and MAPK signaling pathway (Fig. 4B). Therefore, it could be considered that miR-223 may play a role in PDL-derived cells osteogenic differentiation.

**Fig. 5:**
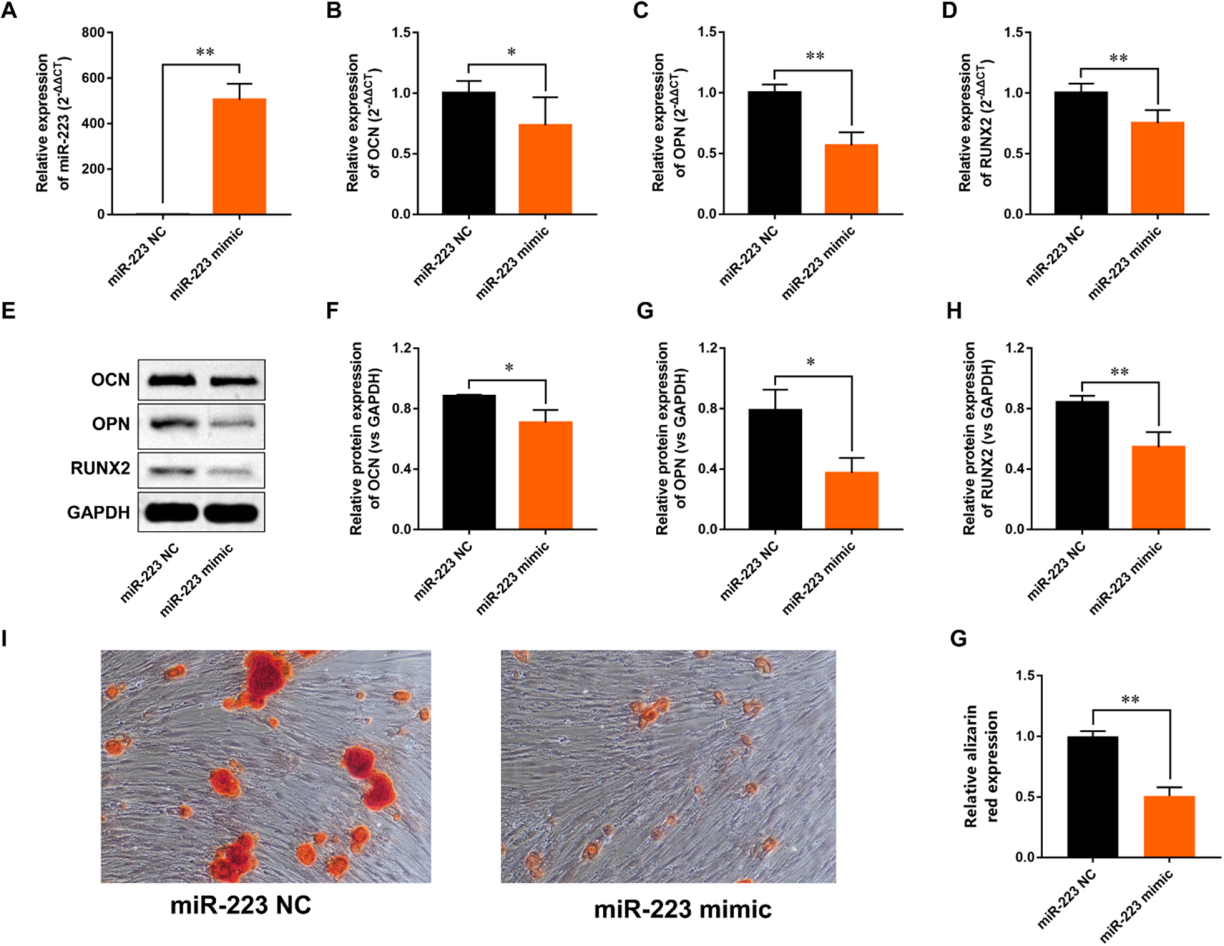
Overexpression of miR-223 inhibits osteogenic differentiation of PDL-derived cells. **A** RT-PCR analysis of miR-223 in PDL-derived cells transfected with miR-223 mimic. **B**–**D** The expressions of osteogenic-specific genes were determined by RT-qPCR method. **E**–**H** The expressions of osteogenic-specific genes were detected by Western blot analysis. **I** Representative images of alizarin red staining. **G** Quantitative analysis of alizarin red staining. All experiments were performed in triplicate, and the bars represent the mean ± SD

To investigate the effects of miR-223 on osteogenic differentiation of PDL-derived cells, we overexpressed miR-223 in PDL-derived cells by administration of miR-223 mimic (Fig. 5A). When transfecting with miR-223 mimic, PDL-derived cells exhibited notably reduced mRNA and protein levels of OCN, OPN and Runx2 (Fig. 5B–H). Alizarin-Red staining analysis indicated that overexpression of miR-223 attenuated the osteogenic differentiation of PDL-derived cells (Fig. 5I–G).

### Prediction of target genes of miR-223

**Fig. 6:**
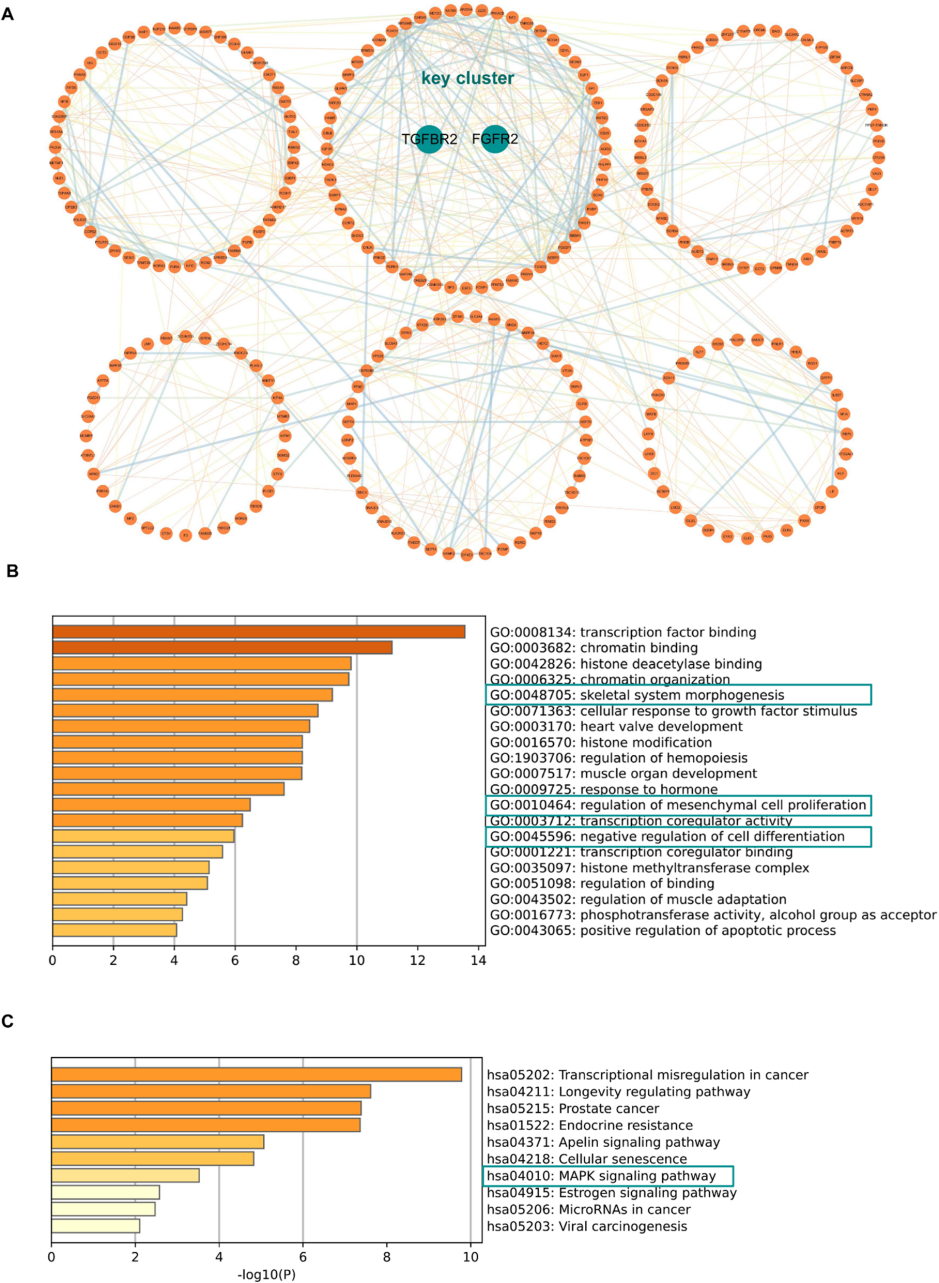
Community discovery clustering network of miR-223 target genes. **A** Six clusters with more than 20 genes were selected. **B** GO terms of key cluster genes. **C** KEGG pathway of key cluster genes

We finally predicted 415 target genes for miR-223, they were listed in Additional file 1: Table S3. The PPI network of predicted target genes was obtained using the STRING database (Additional file 1: Fig. S1). After removing the clusters with less than 20 genes, these genes were divided into six clusters. The largest cluster contained approximately 60 genes, and were defined as key cluster. Remarkably, two important growth factor receptor genes (TGFβR2 and FGFR2) were concentrated in the key cluster (Fig. 6A). GO enrichment results showed that the key cluster was associated with bone morphogenesis, cell proliferation and differentiation (Fig. 6B). The pathways of the key cluster were mainly enriched in MAPK signaling pathway (Fig. 6C).

**Fig. 7:**
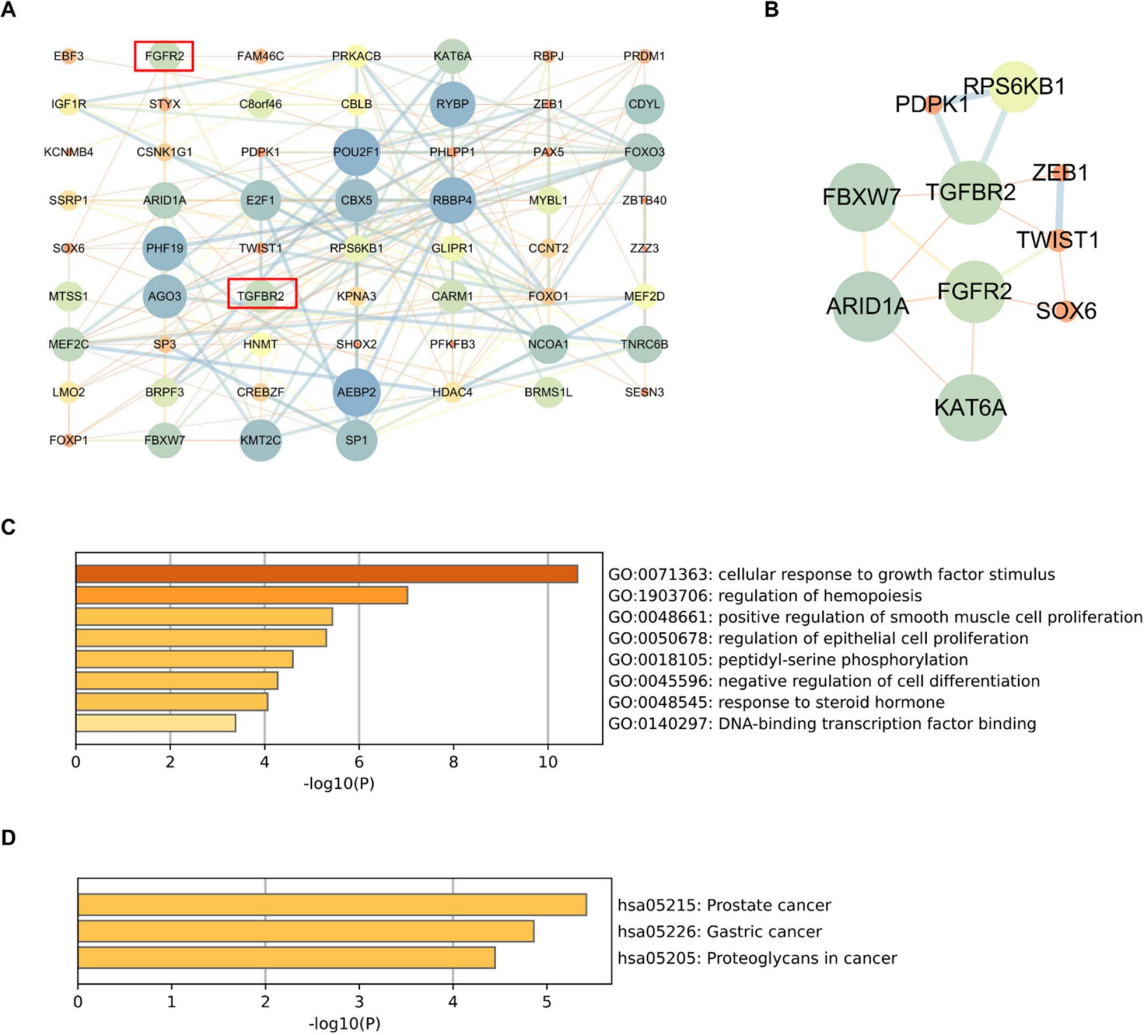
Functional and pathway enrichment analysis of TGFβR2, FGFR2 and they related genes in key cluster. **A** The PPI network of genes in key cluster. **B** The PPI subnetwork centered on TGFβR2 and FGFR2. **C** GO terms of TGFβR2, FGFR2 and they related genes. **D** KEGG pathway of TGFβR2, FGFR2 and they related genes

The PPI network of genes in key cluster showed that TGFβR2 can interact with six genes, and FGFR2 can interact with five genes (Fig. 7A). The PPI subnetwork centered on TGFβR2 and FGFR2 was constructed, and showed that TGFβR2 and FGFR2 can interact with each other through ARID1A, FBXW7 and TWIST1(Fig. 7B). GO and KEGG enrichment results showed that TGFβR2, FGFR2 and they related genes were involved in cellular response to growth factor stimulus, cell proliferation and differentiation (Fig. 7C–D). Taken together, these findings suggest that TGFβR2 and FGFR2 may be the potential miR-223 target genes that influence the osteogenic differentiation of PDL-derived cells.

### MiR-223 directly targets TGFβR2 and FGFR2

**Fig. 8:**
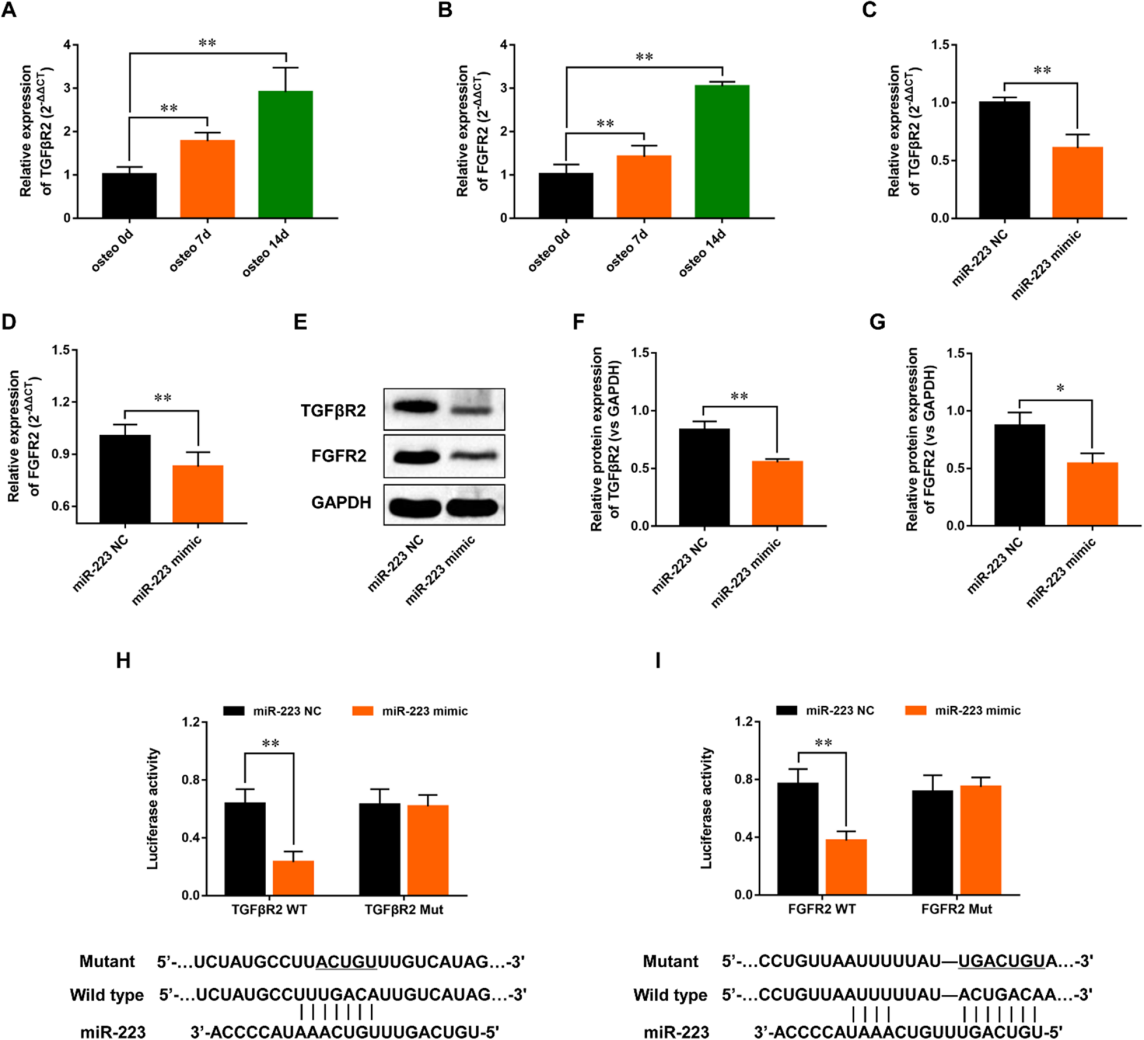
MiR-223 directly targets TGFβR2 and FGFR2. **A**–**B** RT-PCR analysis of TGFβR2 (**A**) and FGFR2 (**B**) in PDL-derived cells after osteogenic differentiation. **C**–**D** RT-PCR analysis of TGFβR2 (**C**) and FGFR2 (**D**) in PDL-derived cells after transfection of miR-223 mimic. **E**–**G** Western blot analysis of TGFβR2 and FGFR2 in PDL-derived cells after transfection of miR-223 mimic. **H**–**I** The effect of miR-223 mimic with TGFβR2 (**H**) and FGFR2 (**I**) 3′UTR-WT/Mut on luciferase activity in PDL-derived cells. All experiments were performed in triplicate, and the bars represent the mean ± SD

TargetScan database predicted that miR-222 directed interactions with the 3′-UTR of TGFβR2 and FGFR2. To explore the role of TGFβR2 and FGFR2 in osteogenic differentiation of PDL-derived cells, their expressions in the PDL-derived cells were measured after the osteogenic differentiation, and we found that TGFβR2 and FGFR2 expressions were up-regulated in a time-dependent manner (Fig. 8A–B). Subsequently, we examined the TGFβR2 and FGFR2 levels in PDL-derived cells after transfection of miR-223 mimic. Compared with the mimic NC group, the gene and protein expressions of TGFβR2 and FGFR2 were markedly decreased in PDL-derived cells transfected with miR-223 mimic (Fig. 8C–G). Dual-luciferase reporter assay showed that the luciferase activity was obviously decreased after co-transfection of miR-223 mimic and WT 3ʹUTR of TGFβR2, while the luciferase activity of MUT 3ʹUTR of TGFβR2 did not change (Fig. 8H). Luciferase activity was decreased in the FGFR2-3’UTR WT group, but was the same in the FGFR2-3’UTR Mut group compared with the negative control (Fig. 8I). These results suggested that miR-223 directly targets both TGFβR2 and FGFR2 that may participate in PDL-derived cells osteogenic differentiation.

### Effects of TGFβR2 on PDL-derived cells osteogenic differentiation

**Fig. 9:**
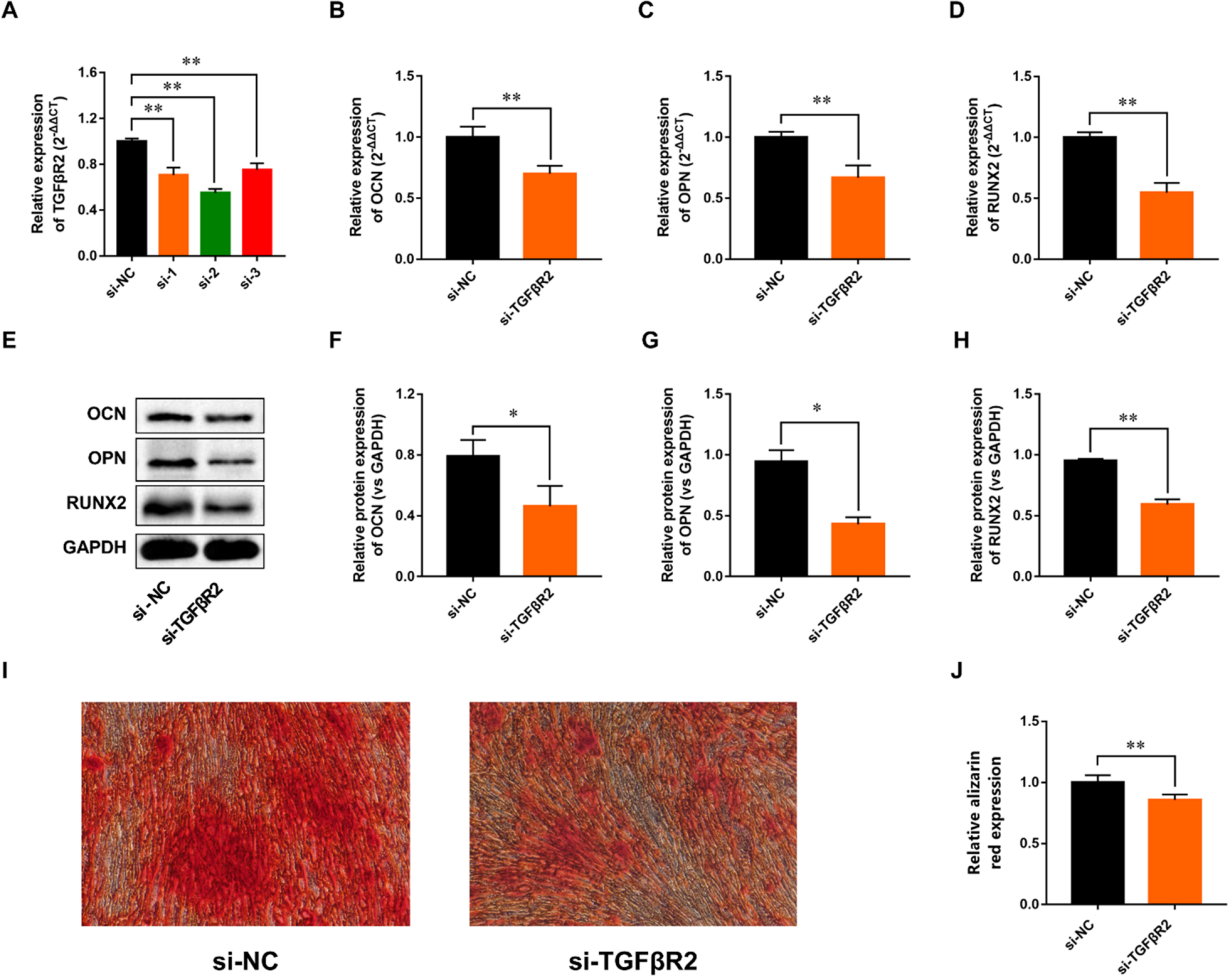
Effects of TGFβR2 on PDL-derived cells osteogenic differentiation. **A** RT-PCR analysis of TGFβR2 in PDL-derived cells transfected with siRNA- TGFβR2. **B**–**D** The expressions of osteogenic-specific genes were determined by RT-qPCR method. **E**–**H** The expressions of osteogenic-specific genes were detected by Western blot analysis. **I** Representative images of alizarin red staining. **G** Quantitative analysis of alizarin red staining. All experiments were performed in triplicate, and the bars represent the mean ± SD

To determine the role of TGFβR2 on PDL-derived cells osteogenic differentiation, we suppressed TGFβR2 using siRNA. Targets of Si- TGFβR2-1, Si- TGFβR2-2 and Si- TGFβR2-3 obviously inhibited the expression of TGFβR2 (Fig. 9A). We choose the target of Si-TGFβR2-2 as subsequent experiment. As shown in Fig. 9B–H, silencing TGFβR2 markedly inhibited the gene and protein expressions of OCN, OPN and Runx2 after osteogenic induction. Assessment of Alizarin-Red staining showed that matrix mineralization was obviously decreased in the PDL-derived cells silencing TGFβR2 compared to the Si-control (Fig. 9I–J). The above results confirm that TGFβR2 plays a key role in PDL-derived cells osteogenesis.

### Effects of FGFR2 on PDL-derived cells osteogenic differentiation

**Fig. 10:**
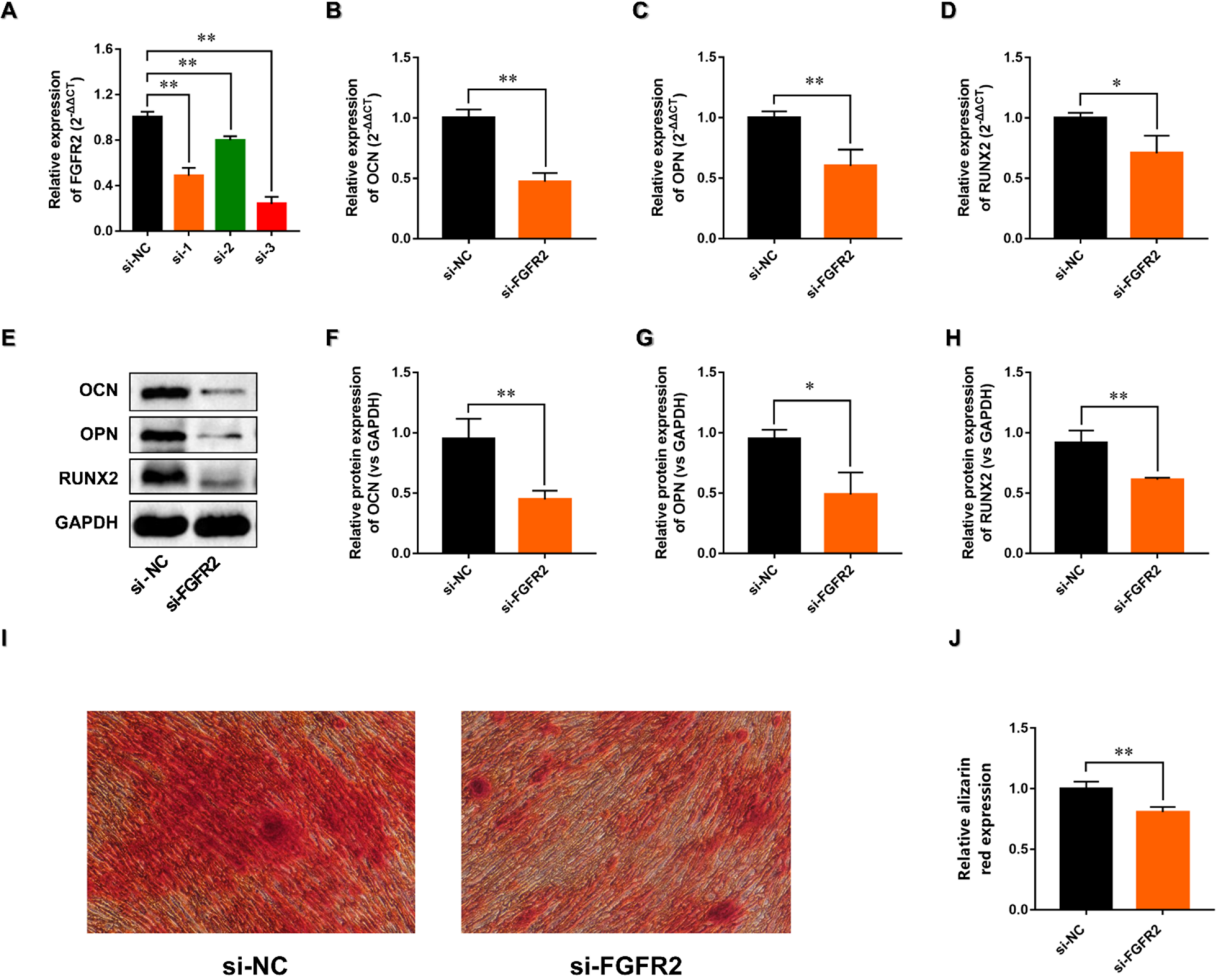
Effects of FGFR2 on PDL-derived cells osteogenic differentiation. **A** RT-PCR analysis of FGFR2 in PDL-derived cells transfected with siRNA-FGFR2. **B**–**D** The expressions of osteogenic-specific genes were determined by RT-qPCR method. **E**–**H** The expressions of osteogenic-specific genes were detected by Western blot analysis. **I** Representative images of alizarin red staining. **G** Quantitative analysis of alizarin red staining. All experiments were performed in triplicate, and the bars represent the mean ± SD

To confirm the function of FGFR2 in the osteogenic differentiation of PDL-derived cells, PDL-derived cells were transfected with siRNA against FGFR2. FGFR2 levels were obviously decreased in PDL-derived cells transfected with Si-FGFR2-1, Si-FGFR2-2 or Si-FGFR2-3, and Si-FGFR2-3 was used in subsequent experiment (Fig. 10A). The gene and protein expressions of OCN, OPN and Runx2 were significantly down-regulated in the FGFR2-knockdown PDL-derived cells compared to the Si-control (Fig. 10B–H). Alizarin-Red Staining showed that si-FGFR2 significantly decreased matrix mineralization in PDL-derived cells (Fig. 10I, J). The results indicate that FGFR2 is involved in PDL-derived cells osteogenesis.

## Discussion

In patients with periodontitis, inflammatory microenvironments have a long-lasting negative effect on the osteogenic differentiation of PDL-derived cells even in ex vivo culture [5]. However, the mechanism remains unclear. The function of miRNAs in MSC osteogenesis has been verified. As a member of miRNAs, miR-223 has been considered as a potential biomarker for periodontitis [18]. However, whether it participates in osteogenic inhibition in periodontitis is unknown. In this study, we found a significant increased expression of miR-223 in inflamed gingival tissues. Additionally, miR-223 expression levels were changed before and after osteogenic induction in PDL-derived cells. More importantly, miR-223 was shown to regulate osteogenic differentiation of PDL-derived cells by targeting growth factor receptor genes, including TGFβR2 and FGFR2. Our results could provide mechanistic insights into the molecular process of osteogenic inhibition in periodontitis.

MiR-223 was first identified in haematopoietic cells [19]. Its abnormal expression in many disorders has been widely studied in recent years [20]. Previously, miR-223 was reported to be involved in the regulation of infections, immune response and several inflammatory disorders [21]. Several microarray studies proved the upregulation of miR-223 among gingival tissue biopsies of periodontitis patients when compared to healthy gingiva [7, 8, 22]. The up-regulated expression of miR-223 was also found in the serum of the periodontitis rat model [23]. Additionally, it was reported that miR-223 was one of the overexpressed miRNAs in both the serum and GCF of periodontitis patients [2]. In accordance, the present study demonstrated significant overexpression of miR-223 in inflamed gingival tissues. These changes may be related to periodontal inflammation induced by oral microbial disorder [24], release of inflammatory mediators [9] and periodontal flap surgery [25]. Furthermore, the expression of miR-223 displayed a significant positive correlation with the clinical parameters in periodontitis group, implying that miR-223 mainly participates in the pathogenesis of periodontitis.

MiR-223 has been proven to be a powerful regulatory RNA in the field of bone biology [20]. Numerous studies documented the potential function of miR-223 in controlling osteoblast differentiation and stimulating differentiation of osteoclasts [15]. Thus, the increased levels of miR-223 in inflamed gingival tissue may play a role in alveolar bone loss, which is an emblem of periodontitis. PDL-derived cells were thought to be an ideal source in alveolar bone repair and play a vital role in maintaining homeostasis of periodontium [26]. Therefore, it will be interesting to study whether dysregulation of miR-223 expression plays a role in the osteogenesis of human PDL-derived cells. In the current study, the expression of miR-223 was noticeably down-regulated during PDL-derived cells osteogenesis, indicating down-regulated miR-223 was beneficial for PDL-derived cells osteogenesis.

The functions of miR-223 in osteoblast differentiation have been reported in previous studies conducted by Guan et al. [10] and Zhang et al. [11]. Guan et al. found that miR-223 was reduced in preosteoblast MC3T3-E1 after osteogenic treatment, and supplementing miR-223 in MC3T3-E1 inhibited osteoblast differentiation [10]. Zhang et al. reported significantly lower miR-223 levels in human BMSCs during osteogenic differentiation, and the inhibition of miR-223 promoted the osteogenic differentiation of BMSCs [11]. Despite these findings, the effect of miR-223 on the osteogenic differentiation of PDL-derived cells, a new population of MSCs derived from gingival tissues, has not been elucidated. Given the downregulation of miR-223 during PDL-derived cells osteogenesis, PDL-derived cells were transfected with miR-223 mimic to further elucidate its roles in PDL-derived cells osteogenesis. Our data indicated that miR-223 overexpression inhibited the osteogenesis of PDL-derived cells, which demonstrated that miR-223 is a negative regulator of PDL-derived cells.

The primary role of miRNAs is the regulation of messenger RNA (mRNA) function by specific binding to target mRNAs. Up to now, more than 20 miR-223 targets have been validated in studies conducted in humans or mice [15]. In our study, bioinformatics analysis and dual luciferase reporter gene assay revealed that miR-223 interacted with two growth factor receptors (TGFβR2 and FGFR2) and inhibited their genes expression. FGFR2 has been identified as miR-223 target in various cells, such as HEK293T cells [27], AD-293 cells [10] and MC3T3-E1 cells [20]. FGFR2 is a critical regulator of osteoblasts. Previous studies showed that activation of FGFR2 signaling enhanced osteoblast differentiation by increasing RUNX2 phosphorylation mediated by MAPK pathway [15]. As shown in our study, the gene silencing of FGFR2 dramatically blocked PDL-derived cells osteogenic differentiation, suggesting that FGFR2 is also involved in miR-223 induced osteogenic dysfunction of PDL-derived cells.

TGFβR2 is the receptor that TGF-β binds directly, and thus it serves as a gatekeeper for the activation of TGF-β downstream signaling [28]. TGF-β is a multi-functional cytokine implicated in the control of cell growth and differentiation [29]. It has previously justified that TGF-β could regulate osteogenesis of periodontal ligament fibroblasts [30]. Previous study also demonstrated the essential role of TGFβR2 in osteogenic periodontal ligament cells during early alveolar bone development, and deletion of TGFβR2 in osteogenic progenitor cells resulted in significant alveolar bone loss [14]. Our results showed that miR‐223 directly binds to the 3′‐UTR of TGFβR2 mRNA, and silencing of TGFβR2 markedly inhibited PDL-derived cells osteogenesis. These results demonstrated that miR-223 could prevent the osteogenic differentiation of PDL-derived cells by suppressing TGFβR2 expression.

## Conclusions

In summary, we have demonstrated that miR-223 can be induced by periodontitis and acts as a negative regulator of PDL-derived cells osteogenesis by targeting two growth factor receptors (TGFβR2 and FGFR2). Our results can provide insight into how to achieve better osteogenic differentiation of PDL-derived cells, and therapeutic inhibition of miR-223 in PDL-derived cells may promote bone formation and even reverse periodontitis.

## Supplementary Information

**Additional file 1. Supplementary Tables and Figures: Table S1.** The primer sequences used in the present study. **Table S2.** The clinical characteristics of the participants. **Table S3**. The target genes of miR-223. **Fig. S1.** The PPI network of miR-223 target genes

## Data Availability

Data used and analyzed during the current study are available from the corresponding author on reasonable request.
