# Supplementary material for: MicroRNA-223 negatively regulates the osteogenic differentiation of periodontal ligament derived cells by directly targeting growth factor receptors
Source: J Transl Med. 2022 Oct 11;20:465. doi: 10.1186/s12967-022-03676-1 (PMC9552407; doi:10.1186/s12967-022-03676-1)
Supplement: Supplementary file 1 — Additional file 1. Supplementary Tables and Figures: Table S1. The primer sequences used in the present study. Table S2. The clinical characteristics of the participants. Table S3. The target genes of miR-223. Fig. S1. The PPI network of miR-223 target genes [file 12967_2022_3676_MOESM1_ESM.docx]

**MicroRNA-223** **negatively regulates the osteogenic differentiation of periodontal ligament derived cells by directly targeting growth factor receptors**

**Running title:** miR-223 inhibits PDLSCs osteogenesis

Zheng Zhang ^1, 2, 3^, Minghui Wang ^4^, Youli Zheng ^4^, Yanmei Dai ^1, 3^, Jiashu Chou ^1^, Xiaowei Bian ^4^, Pengcheng Wang ^5^, Changyi Li ^4^, and Jing Shen ^1,^ ^3^

1. Tianjin Stomatological Hospital, School of Medicine, Nankai University, Tianjin 300041, China

2. State Key Laboratory of Natural and Biomimetic Drugs, Peking University, Beijing 100191, China

3. Tianjin Key Laboratory of Oral and Maxillofacial Function Reconstruction, Tianjin 300041, China

4. The School and Hospital of Stomatology, Tianjin Medical University, Tianjin 300070, China

5. Department of Stomatology, Beijing Shijitan Hospital, Capital Medical University, Beijing 100038, China

Zheng Zhang, Minghui Wang and Youli Zheng contributed equally to this work.

**Correspondence:**

**Jing Shen**, Tianjin Stomatological Hospital, School of Medicine, Nankai University, Tianjin 300041, China. **E-mail:** shenjing611@163.com

**Changyi Li**, The School and Hospital of Stomatology, Tianjin Medical University, Tianjin 300070, China. **E-mail:** lichangyi@tmu.edu.cn

**Pengcheng Wang**, Department of Stomatology, Beijing Shijitan Hospital, Capital Medical University, Beijing 100038, China. **E-mail:** xjztwpc@163.com

**Supplementary table 1**. The primer sequences used in the present study.

| **Species** | **Gene name** | **Primer sequence (5′-3′)** | |
| --- | --- | --- | --- |
|  |  | **Forward primer** | **Reverse primer** |
| **Human** | GAPDH | GCACCGTCAAGGCTGAGAAC | ATGGTGGTGAAGACGCCAGT |
|  | OPN | CGCAGACCTGACATCCAGT | GGCTGTCCCAATCAGAAGG |
|  | OCN | GTAGTGAAGAGACCCAGGCG | CTCCTGAAAGCCGATGTGGT |
|  | Runx2 | AGGCAGTTCCCAAGCATTTCATCC | TGGCAGGTAGGTGTGGTAGTGAG |
|  | FGFR2 | GCATCGCATTGGAGGCTA | TGTGATTGATGGACCCGTATT |
|  | TGFβR2 | GTATCGCCAGCACGATCCCA | CTCGTCATTCTTTCTCCATACAGC |
|  | U6 | GGTCTCGCTTCGGCAGCACA | |
|  | miR-223 | TGTCAGTTTGTCAAATACCCCAAA | |

**Supplementary table 2**. The clinical characteristics of the participants.

|  | **Control group** | **Periodontitis group** | **p-Value** |
| --- | --- | --- | --- |
| **Number of subjects** | 20 | 20 |  |
| **Age (year)** | 42.55 ± 8.66 | 41.95 ± 8.12 | 0.82 |
| **Gender (F/M)** | 10/10 | 9/10 | 1.00 |
| **Mean PI** | 0.40 ± 0.26 | 2.00 ± 0.40 | < 0.001 |
| **Mean GI** | 0.15 ± 0.24 | 1.90 ± 0.38 | < 0.001 |
| **Mean BI** | 0.00 ± 0.00 | 3.08 ± 0.57 | < 0.001 |
| **Mean PD (mm)** | 2.17 ± 0.34 | 4.51 ± 1.11 | < 0.001 |
| **Mean AL (mm)** | 0.34 ± 0.13 | 3.60 ± 1.02 | < 0.001 |

Data are presented as mean ± standard deviation, or number of subjects.

AL, attachment loss; BI, bleeding index; GI, gingival index; PD, pocket depth; PI, plaque index.

**Supplementary table 3**. The target genes of miR-223.

| FBXW7 | PHF19 | WDR77 | SRP19 | MAP4 | TMEM47 | PRKCE | TGFBR3 | RBBP4 | STYX | SESN3 |
| --- | --- | --- | --- | --- | --- | --- | --- | --- | --- | --- |
| RHOB | **ARMC1** | **SSRP1** | **PFN2** | **PHF20L1** | **EPSTI1** | **NRF1** | **ZBTB41** | **LRP12** | **RRAS2** | **PSD3** |
| LELP1 | **SLC25A32** | **CTNNA2** | **XKR6** | **MPZ** | **PKP4** | **CNOT2** | **JMY** | **RORB** | **SH3D19** | **TNRC6B** |
| PTS | **NDP** | **XPR1** | **POU2F1** | **MARCH3** | **SOX11** | **ERC1** | **PLEKHA3** | **SLC35F1** | **SOX6** | **ZIC1** |
| TBC1D17 | **ATP10D** | **DESI2** | **ERO1LB** | **MYBL1** | **PDE4D** | **VAV3** | **CNDP1** | **PFKFB3** | **RAB10** | **TBC1D15** |
| RP11-192H23.4 | **PEX16** | **SHOX2** | **SCARB1** | **EIF4E3** | **RALGPS2** | **KIAA1468** | **ATL2** | **C8orf46** | **CALML4** | **ATG7** |
| WDR62 | **TNNI3K** | **ATP1B1** | **SMARCD1** | **RPS6KB1** | **USP6NL** | **MTMR2** | **TRPV2** | **MBNL1** | **RIMS3** | **FAM83D** |
| LACC1 | **MID1IP1** | **ARPP19** | **MMP16** | **CCT3** | **BAI3** | **MAFB** | **RNF4** | **FOXP1** | **DLEU1** | **MEF2D** |
| GTSF1 | **SEPT8** | **SCAF8** | **RYBP** | **LHX8** | **FGFR2** | **VMP1** | **SECISBP2L** | **HEY2** | **HNMT** | **PHKG2** |
| GALNT18 | **ARPC5L** | **POMP** | **SLC24A2** | **SCN1A** | **PDE3B** | **PLEKHH1** | **KIAA1755** | **NRXN3** | **IFNLR1** | **GPR22** |
| GTPBP8 | **FPGT-TNNI3K** | **NXF1** | **ATP2B1** | **KBTBD6** | **CCNT2** | **TSHZ3** | **ZFX** | **TOP2B** | **NCOA1** | **MCMBP** |
| CCDC149 | **RWDD1** | **ADCYAP1** | **PLAGL2** | **G3BP1** | **SPATA13** | **GPR155** | **DENND5B** | **KMT2C** | **RSBN1L** | **PPP1R15A** |
| C18orf54 | **RCN2** | **PTBP2** | **LRRC19** | **ZEB1** | **OPCML** | **WBP1L** | **GTDC1** | **CARM1** | **PRR14L** | **WDR7** |
| SNX24 | **PURB** | **KLF7** | **FAM46A** | **ZFHX3** | **HMGCS1** | **SDC2** | **AGO3** | **FRMD4A** | **SRGAP3** | **LRRC40** |
| FBXO8 | **CDK17** | **PAX5** | **SPRED1** | **PDS5B** | **LIF** | **MTSS1** | **FBXO28** | **POLR1C** | **TRMT2B** | **AGMO** |
| IL6ST | **MEF2C** | **F3** | **C10orf11** | **RAP2A** | **PRKACB** | **PLCE1** | **ANKFY1** | **WDR43** | **HDAC4** |  |
| PDZD11 | **APC** | **NUP210** | **ACTRT3** | **METAP1** | **ZXDB** | **TSC22D1** | **ZNF395** | **PDPK1** | **GLUL** |  |
| ATP7A | **LYPD6** | **ULK2** | **RASSF4** | **ZZZ3** | **RPS15A** | **IGF1R** | **PIK3C2A** | **PSMA5** | **ZBTB40** |  |
| FOXO1 | **RASA1** | **CLSTN1** | **VHL** | **BRINP3** | **TXLNG** | **CSNK1G1** | **FAM168A** | **ZNF706** | **C17orf75** |  |
| ARMCX1 | **SLC39A1** | **PURA** | **NAA50** | **CNOT1** | **ZBTB10** | **PA2G4** | **CPEB3** | **ELL2** | **UBE2A** |  |
| CYTIP | **PROKR2** | **KAT6A** | **RFTN2** | **NF2** | **TBC1D4** | **DCUN1D3** | **NUDT3** | **E2F1** | **MAP2K6** |  |
| ECT2 | **BRMS1L** | **NLRP3** | **ANKRD17** | **MFSD6** | **RBPJ** | **PAPD5** | **SP1** | **RP11-10A14.4** | **CCDC85C** |  |
| SLC4A4 | **TMEM178B** | **SLCO1A2** | **MMP19** | **FAM98A** | **TAOK3** | **NFIC** | **RC3H1** | **TGFBR2** | **TET3** |  |
| SLC37A3 | **KIAA0226L** | **GPM6B** | **STIM1** | **WASL** | **LONP2** | **EPOR** | **SH2B3** | **ABHD13** | **NOVA2** |  |
| LMO2 | **SLC8A1** | **KPNA3** | **KLF12** | **GABPB2** | **KIF4A** | **CREBZF** | **MAT2B** | **MGA** | **CLPB** |  |
| LAYN | **CRIM1** | **GFPT1** | **COPS2** | **DNAJC6** | **UBE2W** | **RNF217** | **SETBP1** | **TIAL1** | **WDR26** |  |
| INPP5B | **VAMP2** | **MBNL3** | **KLK15** | **FZD4** | **FNBP1L** | **ZXDA** | **SELT** | **HIPK2** | **SNCA** |  |
| RNF145 | **SEPT6** | **NUTF2** | **FOXO3** | **PHIP** | **ZCCHC14** | **ELF2** | **C11orf30** | **TMED7** | **VTI1A** |  |
| ACSL3 | **SREK1** | **SLC23A2** | **ST8SIA3** | **STK39** | **SCN3A** | **AEBP2** | **UHMK1** | **VPS39** | **RP11-315D16.2** |  |
| HSP90B1 | **CTSV** | **MSMO1** | **DOCK2** | **APOBEC4** | **GNA13** | **GRAMD4** | **ZBTB18** | **EBF3** | **ZNF507** |  |
| NFIA | **SRSF10** | **CSPG5** | **ITPR3** | **ZC3H6** | **C14orf142** | **ENPP5** | **KIAA0355** | **FAM46C** | **KCNMB4** |  |
| FAM199X | **PRDM1** | **TMEM64** | **ARID1A** | **MSI2** | **UCP3** | **FBXL4** | **ABCG4** | **ZNF207** | **GRHPR** |  |
| HLF | **SEPT4** | **COPS8** | **SYNCRIP** | **ZBTB4** | **NFASC** | **MAP1B** | **PDS5A** | **MYCBP** | **RGS9BP** |  |
| SP3 | **CBLB** | **HHEX** | **KANSL1L** | **OTUD4** | **UQCC1** | **TSPAN5** | **PHLPP1** | **SRPK2** | **PAX6** |  |
| DNAJB13 | **RNF34** | **INPP4A** | **ELP4** | **SCN2A** | **CEP41** | **SPTLC2** | **CBX5** | **MBOAT2** | **U2SURP** |  |
| SIAH1 | **NUCKS1** | **UBXN1** | **EYA3** | **FAT1** | **ZSCAN12** | **FDX1L** | **ZFP3** | **PCDH12** | **CAND1** |  |
| RGS1 | **ST3GAL1** | **NDNF** | **AKAP1** | **TCERG1** | **MYH10** | **MOSPD1** | **FUBP3** | **CDYL** | **EIF5B** |  |
| ACVR2A | **SORBS1** | **USP42** | **BRPF3** | **ZSWIM7** | **TMEM170A** | **GLIPR1** | **AIFM1** | **FBXO30** | **RBMS2** |  |
| SYAP1 | **NFIB** | **SGMS2** | **SMOC1** | **TMC5** | **ATXN7L1** | **ELK4** | **ABI2** | **TWIST1** | **PKNOX1** |  |
| USP16 | **RERG** | **RAB8B** | **KIAA0226** | **POLR3E** | **RBM20** | **SPPL2A** | **CHUK** | **FAM160B1** | **NLE1** |  |


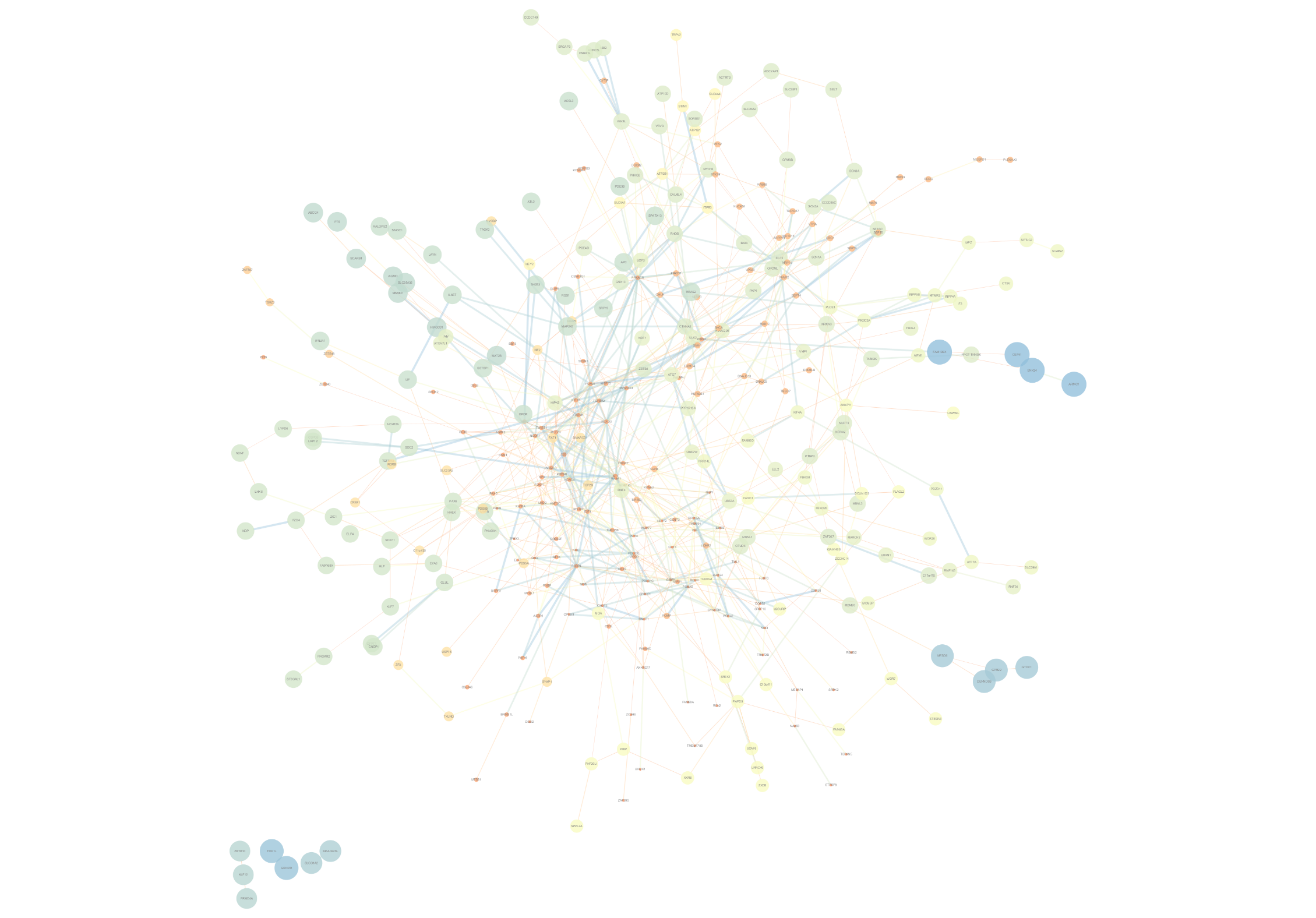


**Supplementary Fig**. 1 The PPI network of miR-223 target genes.
